# Clinical and basic research advances on Jinlong capsule for the prevention and treatment of liver cancer

**DOI:** 10.3389/fphar.2025.1522945

**Published:** 2025-06-09

**Authors:** Liyuan Lv, Simeng Ren, Zunyi Zhang, Hongsheng Lin, Jie Liu

**Affiliations:** ^1^ Department of Hematology and Oncology, Dongzhimen Hospital, Beijing University of Chinese Medicine, Beijing, China; ^2^ Department of Oncology, Guang’anmen Hospital, China Academy of Chinese Medical Sciences, Beijing, China; ^3^ Hepatic Surgery Center, Tongji Hospital, Tongji Medical College, Huazhong University of Science and Technology, Wuhan, Hubei, China

**Keywords:** primary liver cancer, Jinlong capsule, evidence-based research, precision synergistic strategy, prospective research

## Abstract

**Introduction:**

Primary liver cancer, characterized by an insidious onset, rapid progression, high degree of heterogeneity, difficulties in treatment, and a short survival time, poses a significant threat to human health. Jinlong capsule (JLC), an original drug developed in China, is a Chinese patent medicine used to treat liver cancer. Research has demonstrated the antitumor effects of JLC, attributed to its unique preparation process. When used in combination with modern treatment methods, JLC helps in preventing and treating liver cancer recurrence and metastasis, prolonging patient survival, increasing the tumor objective response rate, alleviating gastrointestinal adverse reactions, enhancing survival quality, regulating immune functions of the body, relieving clinical symptoms, and improving patient safety. This study provides a review of clinical and basic research results on JLC.

**Methods:**

We performed literature searches in the Cochrane Library, Embase, PubMed, OVID Scopus, China Biology Medicine, China National Knowledge Infrastructure, VIP, and Wanfang databases for articles published from database inception to December 2023.

**Results:**

Basic research has revealed that the effects of JLC include the inhibition of tumor growth *in vivo* and *in vitro* and immune modulation. The possible mechanisms include inhibiting tumor cell proliferation, promoting tumor cell apoptosis, inhibiting angiogenesis, and modulating cellular immunity.

**Discussion:**

By attuning to the complex biological characteristics of liver cancer, harnessing the unique and unconventional advantages of traditional Chinese medicine, and focusing on clinical needs, we propose directions for future evidence-based research on using JLC in the prevention and treatment of liver cancer. This will contribute to the development of precision synergistic strategies that combine traditional Chinese medicine and modern medicine treatment methods.

## 1 Introduction

Primary liver cancer is the third leading cause of cancer-related death worldwide. A total of 865,269 were diagnosed worldwide in 2022 with a mortality of 757,948 (Bray et al., 2023). Primary liver cancer is currently the fourth most common malignant tumor and the second leading cause of death from tumors in China (Zhou et al., 2019). It includes hepatocellular carcinoma (HCC), intrahepatic cholangiocarcinoma, and combined hepatocellular-cholangiocarcinoma, with HCC accounting for 75%–85% of cases (Sung et al., 2021). In 2020, China recorded 410,038 new cases (accounting for 45.3% of new cases worldwide) of liver cancer and 391,152 liver cancer-related deaths (accounting for 47.1% of deaths worldwide) (World Health Organization, 2020). Approximately 70% of Chinese patients with HCC have middle-to late-stage disease at the time of initial consultation. In China, common treatment methods for liver cancer include hepatectomy (liver resection), liver transplantation, interventional therapy, radiotherapy, systemic therapy, and traditional Chinese medicine (TCM). In the current treatment landscape, the recurrence rate of primary liver cancer at 5 years after surgery is approximately 70% (Yang et al., 2019), and the 5-year overall survival (OS) of patients with liver cancer in China is merely 12.1% (Chinese Society of Liver Cancer, 2021). Therefore, early diagnosis and treatment, multidisciplinary collaboration, and combination therapy are crucial for improving survival and quality of life.

TCM has a long history and demonstrates unique advantages in the prevention and treatment of liver cancer, applicable at different stages of the disease. With the increasing depth of research on the biological characteristics of liver cancer, such as its high degree of heterogeneity and complex tumor microenvironment, the advantages of the holistic concept and multi-target synergy in TCM have become increasingly prominent and form a unique and important component of China’s comprehensive liver cancer prevention and treatment strategy. Jinlong capsule (JLC), an original drug produced from fresh medicinal animal materials by a unique process, is a Chinese patent drug used for the treatment of liver cancer. Since its market entry in 1998, JLC has been widely adopted in clinical practice. Clinical studies have reported that the use of JLC as monotherapy or in combination with modern treatment methods, such as surgery, transarterial chemoembolization (TACE), and radiofrequency ablation (RFA), provides definite advantages in preventing and treating cancer recurrence and metastasis, prolonging patient survival, increasing the tumor objective response rate (ORR), enhancing survival quality, and regulating immune functions of the body, with a favorable safety profile (Li, 2003; Ma et al., 2017; Yan et al., 2021). The use of JLC has been recommended in several Chinese guidelines and consensus statements, including the Chinese Society of Clinical Oncology guidelines on the diagnosis and treatment of primary liver cancer (2020 edition), the expert consensus on the reasonable use of Chinese patent drugs in standardized treatment of cancer pain (2021 edition), the guidelines on the diagnosis and treatment of primary liver cancer promulgated by the National Health Commission of the People’s Republic of China in 2022, and the guidelines for the diagnosis and treatment of integrated Chinese and Western medicine: primary liver cancer (2023 edition) (Chinese Society of Clinical Oncology, 2020; National Health Commission of the People’s Republic of China, 2022; Fan et al., 2021; caim.org, 2023).

**FIGURE 1:**
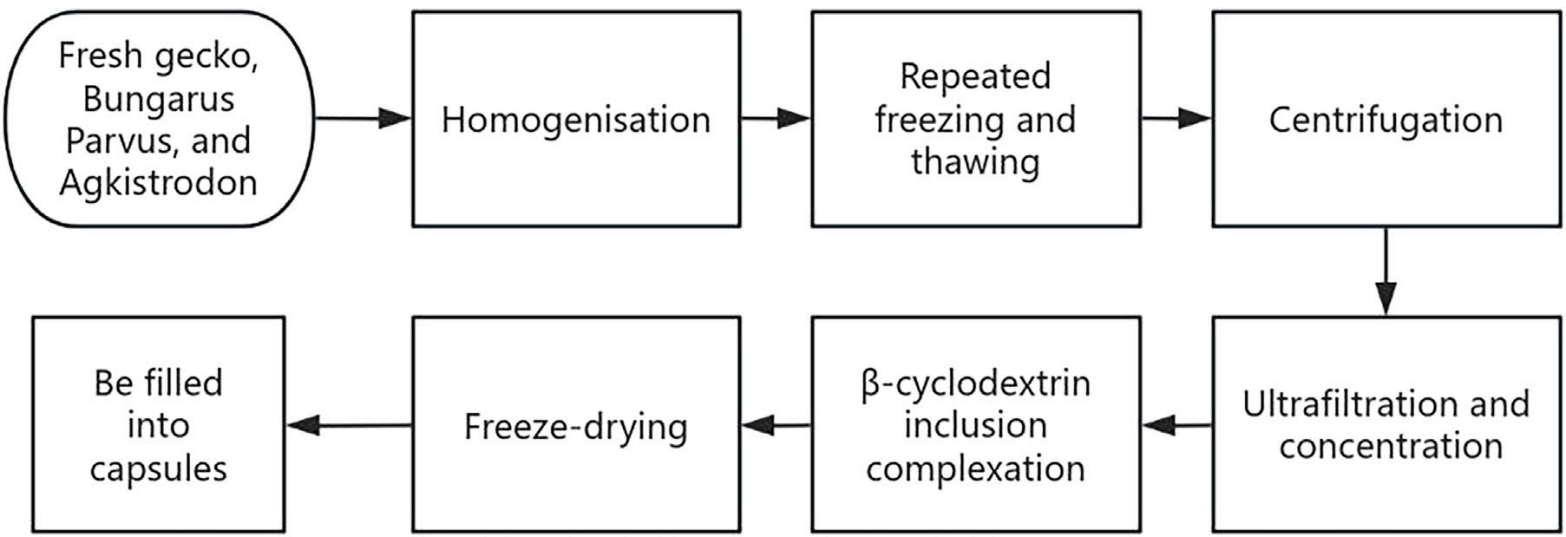
Preparation process flow diagram of Jinlong Capsule(JLC).

The clinical presentation of most patients with primary liver cancer includes fatigue, pain, abdominal distension, loss of appetite, irritability, and hepatosplenomegaly; all of which are symptoms of blood stasis and stagnation syndrome according to the TCM theory. Therefore, treatment should be performed in accordance with the principles of “blood stasis removal and static blood dispersion” and “depression relief and collateral clearing.” JLC was developed based on the TCM theory, is indicated for blood stasis and stagnation syndrome in primary liver cancer, and comprises fresh gecko, Bungarus Parvus, and Agkistrodon. Preparation of JLC involves the extraction, homogenization, repeated freezing and thawing, centrifugation, ultrafiltration, and freeze-drying of fresh medicinal animal materials using modern cryogenic and biochemical separation and extraction techniques (Figure 1). The drug is rich in amino acids, proteins, free monosaccharides, polysaccharides, and small molecules, with up to 98.6% of its components having a molecular weight of <10,000 Da. Its total amino acid and free amino acid contents are 1.5 and 2.6 times, respectively, that of other drugs prepared traditionally. Previous basic research has demonstrated that JLC can inhibit tumor angiogenesis and tumor cell vasculogenic mimicry. This coincides with the TCM theory that blood stasis removal and dispersion promote the normalization of human vasculature and inhibit the formation of twisted and disorganized tumor neo vasculature with coil-like dilations.

In the present study, by incorporating the understanding of the complex biological characteristics of liver cancer achieved by modern medical research with the progress in evidence-based research, we performed literature searches in the Cochrane Library, Embase, PubMed, OVID Scopus, China Biology Medicine, China National Knowledge Infrastructure, VIP, and Wanfang databases for articles published from database inception to December 2023. The Chinese subject headings used in the searches were “金龙胶囊” AND (“肝癌” OR “肝恶性肿瘤” OR “肝肿瘤”), and the English subject headings were “Jinlong capsule” AND (“Hepatic Neoplasms” OR “Cancer of Liver” OR “Liver Neoplasms” OR “Hepatic Cancers” OR “Liver Cancer”). By performing a review of the results of clinical and basic research on JLC and focusing on clinical needs, we aimed to identify approaches for harnessing the unique and unconventional advantages of the TCM and explore the development of precision synergistic strategies that combine the treatment methods of TCM and modern medicine.

## 2 Progress in clinical research on the use of JLC in the prevention and treatment of liver cancer

Surgery is the primary radical treatment method for liver cancer. However, in China, up to 70% of patients have middle-to late-stage liver cancer at their initial consultation. Therefore, the surgical resection rate among Chinese patients is extremely low. The recurrence rate of liver cancer after surgery is high, with a 5-year postoperative recurrence rate of up to 70%. Researchers have actively explored the effects of preoperative neoadjuvant therapy on the enhancement of the radical resection rate among patients with liver cancer. In 2023, the American Society of Clinical Oncology reported using preoperative neoadjuvant hepatic arterial infusion chemotherapy with the FOLFOX regimen in Barcelona Clinic Liver Cancer stage A/B patients with HCC beyond the Milan criteria. The results indicated that the median progression-free survival of patients in the treatment group was 22.7 months, which was longer than that of patients in the direct operation group (10.2 months) (Wei et al., 2023). The use of targeted therapy combined with immunotherapy as conversion therapy in liver cancer has also been investigated. A clinical study on the use of lenvatinib plus an anti-programmed cell death (PD)-1 agent in 56 patients with unresectable middle-to late-stage liver cancer reported a conversion success rate of 55.4% (Zhang et al., 2023). Other studies have examined the effects of postoperative adjuvant therapy on reducing the postoperative recurrence rate. Postoperative adjuvant therapies, such as FOLFOX+ hepatic arterial infusion chemotherapy and atezolizumab combined with bevacizumab, have been implemented in practice; however, there are no widely accepted adjuvant therapy regimens.

For unresectable middle-to late-stage liver cancer, local interventional therapy and systemic medication therapy are commonly adopted treatments. Targeted therapy and immunotherapy further enhance the therapeutic effects based on traditional chemotherapy. Active systemic antitumor treatment can be considered for Child-Pugh class A or B patients (Child-Pugh score ≤7 points). Among the various targeted monotherapy regimens, sorafenib [approved by the Food and Drug Administration (FDA) and National Medical Products Administration (NMPA)] is the first to be used to treat HCC (Llovet et al., 2008; Cheng et al., 2009). A study showed that lenvatinib (approved by the FDA and NMPA) exhibited non-inferiority in the median OS (mOS) compared with sorafenib (13.6 months vs. 12.3 months, hazard ratio = 0.92, 95% confidence interval (CI): 0.79–1.06) (Kudo et al., 2018). Another study reported that donafenib (approved by the NMPA) significantly improved the mOS compared with sorafenib (12.1 months vs. 10.3 months, P = 0.0245) (Qin et al., 2021). Several phase 3 clinical studies have demonstrated that the combined targeted therapy and immunotherapy are superior to sorafenib monotherapy. The results of IMbrave150, a global phase 3 clinical trial, have indicated that atezolizumab plus bevacizumab (approved by the FDA and NMPA) significantly improved the mOS (19.2 months vs. 13.4 months, P < 0.001) (Finn et al., 2020; Finn et al., 2021). In the investigation of the therapeutic effects of immunotherapy alone, the global phase 3 trial HIMALAYA demonstrated for the first time that dual immunotherapy using tremelimumab plus durvalumab (STRIDE regimen, approved by the FDA) was superior to sorafenib. Patients of the STRIDE group showed a significant improvement in the mOS (16.43 months vs. 13.77 months, P = 0.0035) and a lower incidence rate of grade 3 and above treatment-related adverse events (25.8% vs. 36.9%) (Abou-Alfa et al., 2022). However, given that the regimens only benefit limited populations and are costly, the burden of liver cancer remains substantial in China and worldwide.

Therefore, raising the radical resection rate, reducing postoperative cancer occurrence, and enhancing the therapeutic effects in the treatment of middle-to late-stage liver cancer are crucial for achieving the goal of a 15% increase in the 5-year survival rate for all cancers stated in the Outline of the Health China 2030 Plan.

**FIGURE 2:**
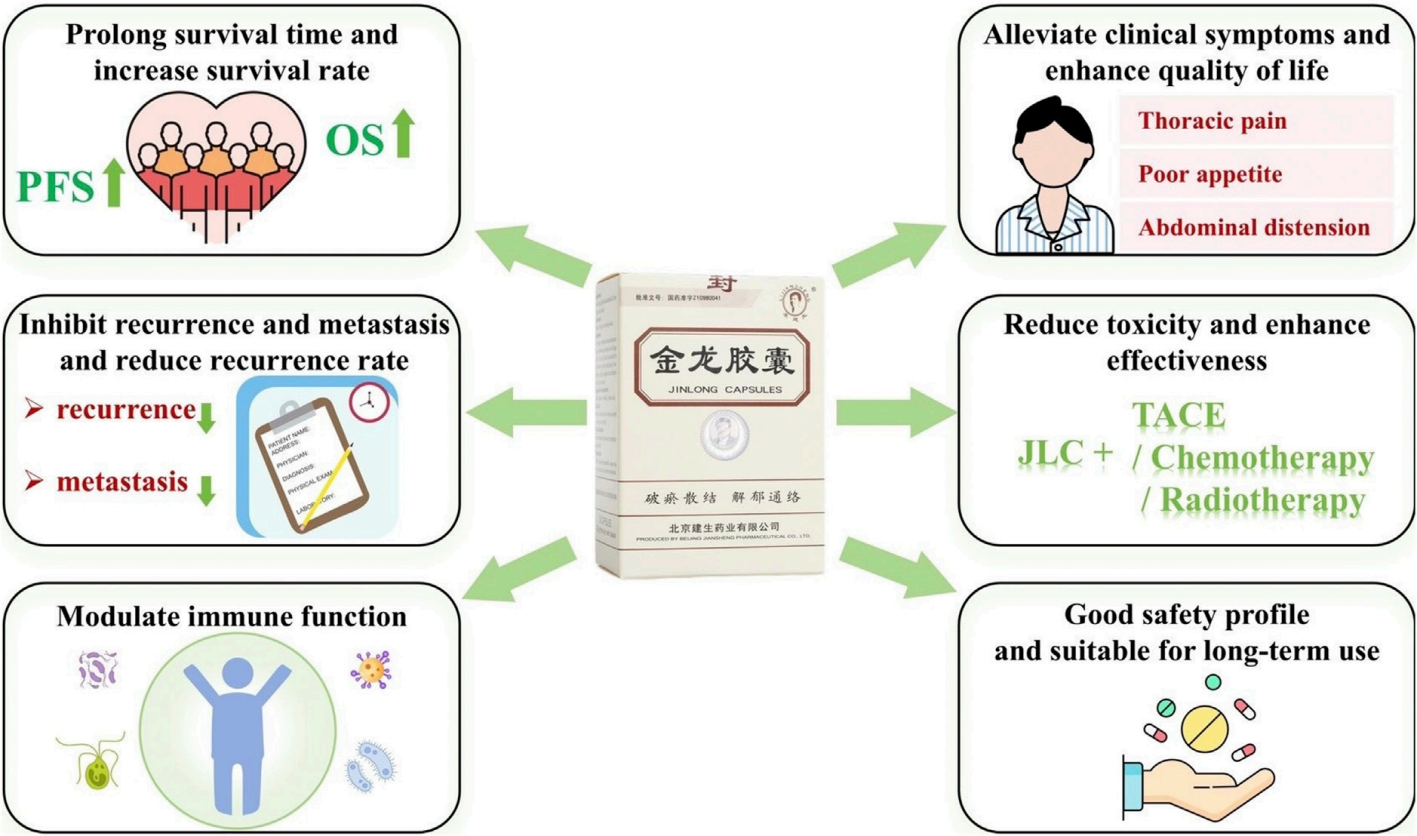
Overview of research on the clinical therapeutic effects of Jinlong capsule (JLC).

**FIGURE 3:**
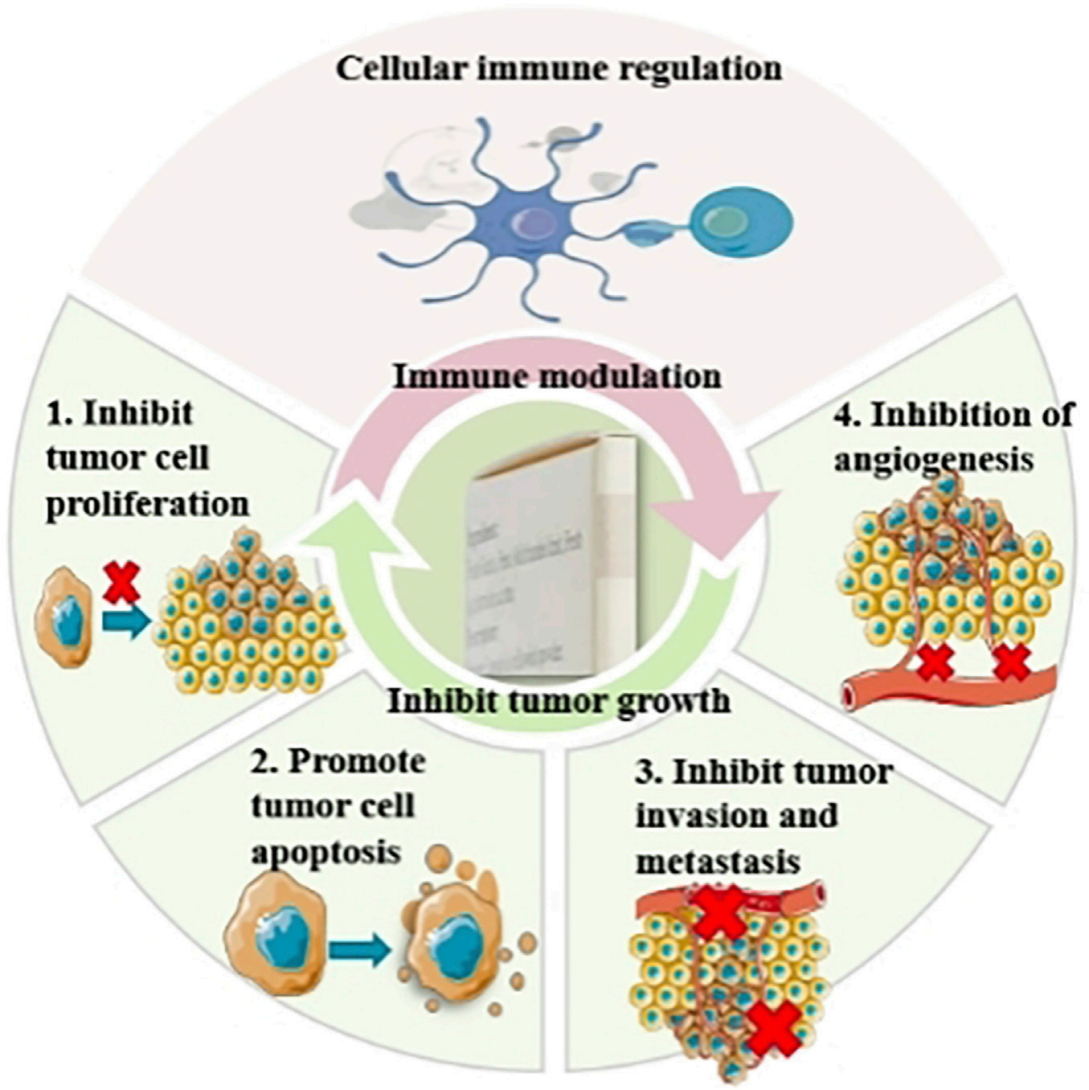
Antitumor mechanisms of Jinlong capsule (JLC). TACE, transarterial chemoembolization.

Clinical studies have indicated the wide use of JLC in various primary liver cancer treatment stages. A total of 68 pertinent clinical research articles were retrieved from the literature, and information on study size and study period of the clinical studies was summarized (Table 1). In which the effects of JLC (Figure 2) combined with surgery, TACE, ablation therapy, chemotherapy, radiotherapy, and targeted therapy were investigated, and the mechanism of action of JLC in anti-tumor was clarified (Figure 3). The main findings of these studies were as follows: (1) JLC combined with surgery prolonged disease-free survival (DFS); (2) JLC combined with TACE increased the tumor ORR and alleviated adverse reactions, such as gastrointestinal reactions; (3) JLC combined with ablation therapy reduced post-ablation liver injury, attenuated symptoms, and strengthened patient immunity, which demonstrated that JLC was a beneficial supplement to RFA; (4) JLC combined with radiotherapy led to an increase in the survival rate; (5) JLC combined with chemotherapy and targeted therapy enhanced the quality of life (QOL) of patients and reduced adverse reactions; and (6) JLC alleviated cancer pain in patients with liver cancer.

**TABLE 1:** The clinical research studies of Jinlong capsule(JLC) for HCC management.

JLC combined with surgery prolonged the DFS rate			
Number	Research scale	Research period	References
1	Single-center Enrollment:122	2 years	Xie et al. (2008b)
2	Single-center Enrollment:90	3 years	Li et al. (2007)

JLC combined with TACE increased the tumour ORR and alleviated adverse reactions			
Number	Research scale	Research period	References
3	Single-center Enrollment:133	2 years	Dong et al. (2008)
4	Single-center Enrollment:60	3 months	Jia et al. (2008b)
5	Single-center Enrollment:63	2 months	Jiang et al. (2013)
6	Single-center Enrollment:147	2 months	Li et al. (2013b)
7	Single-center Enrollment:160	2 months	Liu et al. (2015)
8	Single-center Enrollment:116	3 years	Meng, (2016b)
9	Single-center Enrollment:60	2 months	Wang et al. (2009)
10	Single-center Enrollment:62	1 month	Xie et al. (2003)
11	Single-center Enrollment:70	5 weeks	Yang et al. (2013b)
12	Single-center Enrollment:60	3 months	Yuan et al. (2013b)
13	Single-center Enrollment:224	3 years	Zhang et al. (2005b)
14	Single-center Enrollment:58	2 years	Zheng et al. (2018)
15	Single-center Enrollment:100	2 years	Zheng et al. (2007)
16	Single-center Enrollment:71	1 year	Chen et al. (2007)
17	Single-center Enrollment:224	3 years	Liang et al. (2005)
18	Single-center Enrollment:64	6 months	Zhao and Nie (2007)
19	Single-center Enrollment:26	2 years	Qi and Li (2007)
20	Three Centers Enrollment:64	2 months	Zeng (2007)
21	Single-center Enrollment:43	2 years	Wei et al. (2007)
22	Single-center Enrollment:54	3 years	Wen et al. (2010)
23	Single-center Enrollment:100	5 months	Huang and Yang (2007)
24	Single-center Enrollment:60	1 month	Yang (2007)
25	Single-center Enrollment:48	2 months	Zhang et al. (2008)
26	Three Centers Enrollment:61	2 months	Zeng et al. (2010)
27	Single-center Enrollment:45	2 years	Long et al. (2010)
28	Single-center Enrollment:9	3 years	Li (2010)
29	Single-center Enrollment:60	4 months	Zeng et al. (2012)
30	Single-center Enrollment:151	4 years	Zhang et al. (2012)
31	Single-center Enrollment:82	1 month	Huang et al. (2013)
32	Two Centers Enrollment:46	3 months	Yang et al. (2014)
33	Single-center Enrollment:64	3 months	Huang and Pu (2015)
34	Single-center Enrollment:206	5 months	Dong (2015)
35	Single-center Enrollment:98	2 months	Fan et al. (2019)
36	Single-center Enrollment:148	4 months	Wang et al. (2022)
37	Single-center Enrollment:82	1 month	Huang et al. (2015)
38	Single-center Enrollment:60	2 months	Zeng et al. (2014)
39	Single-center Enrollment:46	2 years	Ma (2017)
40	Single-center Enrollment:98	2 months	Liu et al. (2021)
41	Single-center Enrollment:42	2 years	Sun et al. (2008)

JLC combined with RFA, chemotherapy, and radiotherapy led to targeted tumour stabilisation, symptom improvement, and enhanced immunity			
Number	Research scale	Research period	References
42	Single-center Enrollment:76	3 years	Liu et al. (2013)
43	Single-center Enrollment:27	6 weeks	Zhang (2012)
44	Single-center Enrollment:85	3 years	Wang and Yang (2013b)
45	Single-center Enrollment:30	1 year	Xie et al. (2019a)
46	Single-center Enrollment:94	3 years	Ye et al. (2008)
47	Single-center Enrollment:64	1 month	Fan et al. (2015)
48	Single-center Enrollment:60	2 months	Lu et al. (2015)
49	Single-center Enrollment:96	1 year	Yin et al. (2008)
50	Single-center Enrollment:52	1 year	Xiao et al. (2009)
51	Single-center Enrollment:39	10 weeks	Xu (2015)
52	Single-center Enrollment:68	18 weeks	Wang (2015)
53	Single-center Enrollment:72	10 weeks	Li and Mao (2016)
54	Thirty Centers Enrollment:499	4 years	Li (2003)
55	Single-center Enrollment:62	2 months	Sun and Zhou (2010)
56	Single-center Enrollment:62	6 months	Cai (2011)
57	Single-center Enrollment:37	13 weeks	Lu et al. (2012)

JLC alleviated cancer pain in older patients with late-stage malignancies and improved symptoms of abdominal distension			
Number	Research scale	Research period	References
58	Single-center Enrollment:82	4 months	Xie et al. (2013)
59	Single-center Enrollment:62	3 years	Huang (2006)
60	Single-center Enrollment:40	1 year	Tang and Cao (2002)
61	Single-center Enrollment:50	9 months	Zhu (2003)
62	Single-center Enrollment:128	1 year	Sun et al. (2006)
63	Single-center Enrollment:52	3 months	Xiong et al. (2010)
64	Single-center Enrollment:44	3 months	Zhang et al. (2012)
65	Single-center Enrollment:40	1 month	Tang et al. (2015)
66	Single-center Enrollment:82	10 years	Cheng (2018)
67	Single-center Enrollment:8	10 months	Li et al. (2005)
68	Single-center Enrollment:30	40 months	Shi (2000)

### 2.1 JLC combined with surgery prolonged the DFS rate

The postoperative recurrence rate of liver cancer is associated with the preoperative presence of microscopic, disseminated lesions or multicentric occurrence of the disease. Xie et al. (2008a) performed a study on 122 patients with primary liver cancer whose tumors could be completely resected (stage 1: 68 patients; stage 2: 54 patients). Patients in the treatment group received JLC at 1 g three times a day and were observed for 6 months, whereas the control group was administered fluorouracil (5-FU) starting from 4 weeks after surgery by intravenous infusion at a dose of 330 mg/m^2^ once every 4–6 weeks. The results indicated that the treatment group had a mOS of 15.0 months, which was significantly longer than that of the control group (10.7 months, P = 0.01), and exhibited an improvement in QOL.

### 2.2 JLC combined with TACE increased the tumor ORR and alleviated adverse reactions, such as gastrointestinal reactions

TACE is currently one of the most used non-surgical treatment methods in liver cancer. Research has shown that combining TACE with TCM enhanced patient survival and QOL while reducing the occurrence of adverse reactions, such as gastrointestinal reactions and myelosuppression (Xie et al., 2003; Zhang et al., 2005a; Dong et al., 2008; Jia et al., 2008a; Wang et al., 2009; Jiang et al., 2013; Li et al., 2013a; Yang et al., 2013a; Yuan et al., 2013a; Liu et al., 2015; Meng, 2016a; Zheng et al., 2018). A meta-analysis by Yan et al. (2021) that included 19 randomized controlled studies on the use of JLC combined with TACE for the treatment of primary liver cancer (1,740 cases) revealed that the combination therapy group had a higher ORR [Odds ratio (OR) = 2.23, 95% CI: (1.78–2.80), P < 0.001] and a higher Karnofsky Performance Status score [OR = 2.59, 95% CI: (1.86–3.60), P < 0.001]. Combination therapy with JLC and TACE also led to a reduction in adverse reactions, such as gastrointestinal reactions [OR = 0.43, 95% CI: (0.24–0.78), P = 0.005] and leukopenia [OR = 0.36, 95% CI: (0.27–0.49), P < 0.001] compared with the control group.

### 2.3 JLC combined with RFA, chemotherapy, and radiotherapy led to targeted tumor stabilization, improved symptoms, and enhanced immunity

Liu et al. (2013) performed observations of patients who underwent sequential treatment with JLC following RFA and compared them with patients who underwent RFA monotherapy. Treatment effectiveness was 53.84% in the observation group and 42.86% in the control group, with a statistically significant difference between the two groups (P < 0.05). Therefore, it is evident that JLC was capable of tumor stabilization, inhibiting tumor progression, improving survival quality, alleviating symptoms, strengthening patient immunity, and reducing post-RFA liver injury, thereby serving as a beneficial supplement to RFA. A study by Zhang (2012) involving 27 patients with lung metastases from primary liver cancer reported that the group that received chemotherapy with concomitant oral administration of JLC had greater short-term clinical benefit, a higher proportion of patients with reduced serum alpha-fetoprotein levels, better physical conditions, lower incidences of grade 3/4 leukopenia, and significantly higher CD4/CD8 count compared with patients who received chemotherapy alone. Wang and Yang (2013a) found that the 1- and 3-year OS of the combined three-dimensional conformal radiation therapy and JLC group were 74.4% (32/43) and 34.9% (15/43), which were higher than those of the radiation monotherapy group [66.7% (28/42) and 16.7% (7/42), P = 0.046]. Xie Y. L. et al. (2019) performed follow-up observations of patients who received combination therapy with apatinib (treatment group) and JLC and those who received apatinib alone (control group). At 3 months, 6 months, and 1 year after treatment, the treatment group showed improvements in alpha-fetoprotein, imaging measurements of tumor changes, and ascites compared with the control group. The treatment group also exhibited a better short-term QOL, lower incidence of adverse reactions, higher remission rate, and prolonged survival (P < 0.05).

### 2.4 JLC alleviated cancer pain in older patients with late-stage malignancies and improved abdominal distension symptoms

A study by Xie et al. (2013) on 82 older patients with late-stage malignancies showed that compared with the control group, treatment with JLC led to the alleviation of cancer pain and an improvement in the Karnofsky Performance Status score. Therefore, the 2021 edition of the expert consensus on the reasonable use of Chinese patent drugs in the standardized treatment of cancer pain recommends the use of JLC to relieve thoracic pain caused by cancers such as primary liver and pancreatic cancers. Huang (2006) performed clinical observations of 62 patients with late-stage liver cancer who received a combination of JLC and syndrome-based medication therapy, paying particular attention to symptoms such as thoracic pain and abdominal distension. The results indicated that the complete disappearance of symptoms occurred in 20 patients, and a partial improvement in the symptoms occurred in 30 patients, resulting in a high symptom improvement rate of 80.7%. This demonstrates that JLC, combined with syndrome-based medication therapy, can alleviate thoracic pain, abdominal distension, and other clinical symptoms associated with primary liver cancer.

## 3 Progress in basic research on the use of JLC in the prevention and treatment of liver cancer

Basic research on the use of JLC (Table 2) in the treatment of liver cancer has primarily focused on the effects of JLC on tumor growth inhibition and immune modulation. Potential mechanisms include inhibiting tumor cell proliferation, promoting tumor cell apoptosis, inhibiting tumor invasion and migration, inhibiting angiogenesis, and modulating cellular immunity.

**TABLE 2:** The basic research studies of Jinlong capsule(JLC) for HCC management.

Number	Mechanism of action	References
1	Jinlong Capsule possesses certain potential for chemotherapy resistance reversal and chemosensitization in both 5-Fu-resistant human hepatocellular carcinoma cells Bel7402/5-Fu and their parental cells Bel7402 towards vincristine, with demonstrated cell selectivity and chemotherapeutic drug selectivity	Qu et al. (2014)
2	Jinlong Capsule significantly inhibits the adhesion, migration, and invasion capabilities of MHCC97H hepatocellular carcinoma cells, thereby suppressing tumor cell metastasis	Li et al. (2011)

### 3.1 Inhibition of tumor growth

An *in vivo* experiment was performed on nude mice inoculated with human liver carcinoma (HepG2) cells. The results indicated that the tumor inhibition rate was 37.6% after 4 weeks of drug administration. In another study that investigated the effect of JLC administration for 9 days on Kunming mice inoculated with the H22 mouse hepatoma cell line, the 37 g crude drug/kg dose group exhibited an average tumor inhibition rate of 44.4%. Xie Y. Z. et al. (2019) found that a high dose of JLC (1500 mg/kg) led to a tumor inhibition rate of 61.90% in nude mice receiving intracranial implantation of a U87-RFP glioma. Liu et al. (2001) performed interventions twice with JLC in a high metastatic tumor model of murine cervical carcinoma (U14) and reported that the inhibition rates of local tumor recurrence were 54.8% and 66.3% for the two interventions, while the inhibition rates of metastasis were 50% and 54%. *In vitro* experiments revealed that the main antitumor mechanisms of JLC were as follows.

#### 3.1.1 Inhibition of tumor cell proliferation

Li et al. (2018) found that JLC significantly inhibited the proliferation rate of the MGC-803 and BGC-823 gastric cancer cell lines in a concentration- and time-dependent manner. A study by Li (2012) reported that JLC provided good inhibitory effects on the proliferation of the BxPC-3 human pancreatic cancer cell line. Lü et al. (2010) performed *in vitro* culture of lung adenocarcinoma cells and used the cell counting method to observe the influence of different concentrations of JLC on cell growth. The results showed that the growth curve of the drug administration group was less steep compared with the control group and was significantly positively correlated with drug dose. This demonstrates that JLC exerted a significant and dose-dependent inhibitory effect on the proliferation of lung adenocarcinoma cells.

#### 3.1.2 Promotion of tumor cell apoptosis

Zhao et al. (2002) found that JLC induced apoptosis of human leukemia (HL)-60 cells mainly during the S phase of the cell cycle. HL-60 cells treated with three different dose levels of JLC exhibited apoptotic rates of 17.05%, 40.06%, and 53.45% at 72 h after treatment, with both early and late apoptosis observed in the cells. In contrast, the apoptosis rate of the control group was only 6.97%. Li et al. (2018) reported that JLC promoted apoptosis in MGC-803 and BGC-823 human gastric cancer cell lines and blocked cell cycle progression at the S and G2/M phases. An investigation of the pertinent mechanisms revealed that this effect was mainly associated with reducing the protein expression levels of Bcl-2 and surviving.

#### 3.1.3 Inhibition of angiogenesis

Existing literature has reported that the mechanisms by which JLC inhibits tumor angiogenesis are mainly related to the expression of matrix metalloproteinases and vascular endothelial growth factor (VEGF). Using an animal model and molecular biology experiments, Li (2011) demonstrated that the protein expression levels of VEGF, matrix metalloproteinase-2, and matrix metalloproteinase-9 in mouse tumor tissue were significantly lowered after treatment with JLC. Liu et al. (2016) reported that high, moderate, and low doses of JLC interfered with angiogenesis and downregulated the mRNA and protein expression of Mig-7 in the HCT116 colon cancer cell line.

#### 3.1.4 Inhibition of tumor cell invasion and migration

Shi et al. (2019) performed *in vitro* experiments with the A172 and U251 human glioma cell lines and found that JLC inhibited tumor cell invasion and migration. An increase in the JLC concentration led to a significant dose-dependent decrease in the number of cells involved in invasion and migration. Further investigation of the mechanisms of action revealed that JLC exerted inhibitory effects on tumor cell invasion and migration by inhibiting the expression of phosphorylated mammalian target of rapamycin and phosphorylated S6. Many genes are associated with the growth, invasion, and adhesion of tumor cells, among which vanin 1 exhibited the most significant upregulation. Huang et al. (2014) established an animal model of *in situ* brain glioma and discovered that the vanin 1 expression level of the low-dose JLC group was 48.34 times that of the model group. This result suggests that JLC effectively inhibited intracranial tumor growth in nude mice, with vanin 1 as a potential target.

### 3.2 Immune modulation

In an *in vitro* study by Xu et al. (2005), JLC increased CD3^+^ cells, CD4^+^ cells, and the CD4+/CD8+ ratio in immunosuppressed animals. This indicates that JLC significantly countered immunosuppression induced by intraperitoneal injection of cyclosporin A and promoted the recovery of immune function. In clinical studies investigating the effects of JLC combined with TACE and chemotherapy, the treatment groups exhibited an increase in the CD4+/CD8+ ratio, which was indicative of improved cellular immune function.

## 4 Discussion

According to TCM, the liver governs the unclogging and deflation of qi and emotions and favors free and unobstructed movement. The loss of the unclogging and deflation function in the liver will lead to stagnation of liver qi, which leads to blood stasis. Long-term blood stasis accumulates beneath the ribs, becomes toxic, and develops into cancerous tumors. Therefore, stasis-induced toxicity, toxicity-induced pathological changes, and stasis-toxicity intermingling are the core pathogenic mechanisms of liver cancer. In approximately 40% of patients with primary liver cancer, the edges of the tongue exhibit green, dark purple, linear, strip-like, spot-like, or irregularly shaped patches with ecchymoses and petechiae. These patches, which possess distinct borders and may be raised above the tongue surface, are known as “lines of ganyin” (Wang and Wang, 2014). They serve as rough indicators for the investigation of middle-to-late-stage primary liver cancer and can indicate disease severity and prognosis. Hui et al. (2023) systematically searched the literature on TCM syndrome differentiation of primary liver cancer and retrieved 90 articles (10,304 patients) that fulfilled the search criteria. The results revealed that the qi stagnation and blood stasis syndrome was the most common TCM syndrome among the patients (20.77%, 2,140 patients). Therefore, the use of the blood stasis syndrome as a key research component in exploring features that benefit populations can be considered. As of the end of 2023, 77 clinical studies on the application of JLC in other types of cancer have been published. The cancer types include stomach cancer, pancreatic cancer, lung cancer, breast cancer, colorectal cancer, nasopharyngeal carcinoma, cervical cancer, and brain glioma, and the effects include the prevention of recurrence and metastasis, reduction of toxicity and enhancement of effectiveness, and immune modulation (Huang et al., 2016; Li and Mao, 2016; Zou et al., 2016; Dong et al., 2018; Lai et al., 2021; Wen et al., 2021; Pei and Wang, 2022; Wu and Cheng, 2022). TCM treatment is performed based on syndrome differentiation, and the concept of treating different diseases with the same therapy if they belong to the same syndrome is also widely applied. JLC is used in treating multiple types of solid tumors, which is akin to the important role served by anti-angiogenesis therapy and immune checkpoint inhibitors in various cancers, such as stomach cancer, lung cancer, and liver cancer. Blood stasis syndrome is the common target among these treatment methods, suggesting the presence of common mechanisms in the prevention and treatment of multiple types of solid tumors by JLC. Therefore, further exploring the material basis and molecular phenotype characteristics underlying blood stasis syndrome may be worthwhile.

Given the clinical needs of liver cancer, which include the resolution of the issues of a low surgical resection rate and high postoperative recurrence rate, it is imperative that neoadjuvant and adjuvant regimens consisting of JLC are explored to enhance the pathological complete response rate and surgical resection conversion rate. In completed studies on the use of JLC as a postoperative adjuvant therapy, the endpoint was the median survival, and the control group had received chemotherapy with 5-FU. Currently, studies on postoperative adjuvant therapies for liver cancer are no longer solely focused on chemotherapy; they have further delved into the exploration of targeted therapy, anti-angiogenesis therapy, and immune therapy, with most study endpoints being relapse-free survival or DFS. Therefore, indicators such as relapse-free survival and DFS can be set as study endpoints in future research. For patients at high risk of recurrence, the combination of JLC with neoadjuvant therapy, TACE, lenvatinib, or atezolizumab can be investigated in studies on postoperative adjuvant therapies, with the aim of achieving better therapeutic effects based on current treatment methods. In addition, considering that the main functions of JLC are “blood stasis removal and static blood dispersion” and “depression relief and collateral clearing,” future clinical studies may also be focused on populations with blood stasis syndrome or the use of patients with blood stasis syndrome as a separate stratification factor for outcome analysis, to identify the true benefiting populations.

The progress of new drug development and clinical research has led to the emergence of various first-line treatment strategies for unresectable or metastatic HCC. Given the clinical needs related to the limited therapeutic effects of combination therapy on mid-to-late-stage liver cancer and the toxic side effects of therapies, the exploration of combination treatment modalities involving the use of JLC with TACE, chemotherapy, radiotherapy, targeted therapy, and RFA therapy will be of great significance for improving the outcomes of patients with liver cancer. Most previous studies on unresectable or metastatic HCC have merely investigated the combination of JLC with TACE. In these studies, an emphasis has also been placed on the short-term therapeutic effects, with the tumor ORR mainly adopted as the study endpoint and less attention given to the mOS. Therefore, OS may be the primary efficacy endpoint in future study designs. In a clinical study on icaritin, the benefiting population was defined as patients with tumor necrosis factor-alpha levels <2.5 pg/mL and interferon-gamma levels ≥7.0 pg/mL, suggesting that icaritin may be more beneficial for patients with HCC with concomitant hepatitis B virus and/or hepatitis C virus infections (Sun et al., 2021). Given that JLC is a dominant drug for blood stasis removal and static blood dispersion and that the incidence of the blood stasis syndrome in patients with concomitant viral hepatitis and cirrhosis is relatively high in clinical practice, it is possible that JLC would be more useful for patients with liver cancer and hepatitis or cirrhosis. The characteristics of immunological markers or cytokines in these populations should be studied further. In future studies, researchers may perform detailed investigations of these specific populations or include such populations as a stratification factor for analyses within large, comprehensive studies. Besides research on the use of JLC in postoperative adjuvant therapies, it is also worthwhile to conduct clinical studies related to the combined use of JLC with targeted drugs or immunotherapy to further enhance existing therapeutic effects.

The inhibition of tumor angiogenesis serves as the mechanism of action in most targeted drugs currently used for the treatment of liver cancer, including sorafenib, lenvatinib, and bevacizumab. The occurrence and development of liver cancer is closely associated with tumor angiogenesis. VEGF-mediated angiogenesis is a major driving factor for immune evasion in tumors. Upon binding with VEGF receptor-2 (VEGFR2), VEGF can promote endothelial cell proliferation and migration and induce vascular changes in liver cancer, thereby promoting liver cancer cell growth (Lan et al., 2024). PD-1 is a checkpoint molecule that is expressed on the surfaces of natural killer T cells, B cells, dendritic cells, monocytes/macrophages, CD4^+^ T cells, and CD8^+^ T cells. Interactions between PD-1 and its ligand, PD-L1, inhibit T cells' activation, proliferation, and effects. Under physiological conditions, PD-1, PD-L1, and PD-L2 activate T cells during peripheral tolerance. This leads to the inhibition of immune tissue damage caused by self-reactive T cells, thereby preventing autoimmunity. Under pathological conditions, activation of the PD-1/PD-L1 pathway can induce T cell deactivation, apoptosis, and exhaustion; modulate the differentiation of CD4^+^ T cells into Treg cells; and suppress immune responses. Solid tumors can hijack the PD-1/PD-L1 axis, induce immunosuppression, and evade T cell receptor recognition. It has been demonstrated that blockade of the PD-1/PD-L1 axis causes reversal of the immunosuppressive phenotype, activates the adaptive immune system, triggers tumor antigen recognition, activates cytotoxic CD4^+^ and CD8^+^ T cells, and enhances antitumor efficacy. Therefore, monoclonal antibodies targeting PD-1/PD-L1 can exert antitumor effects (Liu et al., 2023). Sintilimab and atezolizumab are PD-1/PD-L1 inhibitors commonly used in the clinical treatment of liver cancer. Good therapeutic effects have been achieved with the combined use of anti-angiogenic agents and PD-1/PD-L1 inhibitors in clinical treatment. This further demonstrates the complex biological characteristics of liver cancer and suggests that combined treatments with multiple targets may lead to better clinical therapeutic effects. JLC is characterized by low toxicity, multiple targets, and multiple pathways; however, its detailed mechanisms of action have not yet been fully elucidated as previous basic research lacks research methods and depth. For instance, the investigation of immune modulation has been largely limited to a single indicator, namely CD4+/CD8+. Tumor immunity encompasses many types of negative immune regulators released in the tumor microenvironment, including PD-1, lymphocyte-activation gene −3, and indoleamine 2,3-dioxygenase (Chen et al., 2024), which should be further investigated. Besides improving the tumor environment, JLC also exerts effects on tumor cells, which should not be underestimated. Tumor stem cells, characterized by self-renewal and an unlimited proliferative capacity, are highly associated with liver cancer recurrence and metastasis (Jiang et al., 2024; Chen et al., 2024). Therefore, the exploration of the mechanisms of action of JLC on tumor stem cells also serves as a basis for preventing recurrence and metastasis from the source.

Primary liver cancer possesses high heterogeneity and clinical complexity. Therefore, the prognosis of patients with liver cancer remains poor, and the current treatment landscape requires further improvement. JLC has been used in clinical practice for years and has demonstrated definite therapeutic effects. Previous clinical studies have reported that the combined use of JLC with modern medical therapies enables the synergistic enhancement of treatment effects, and basic research has indicated the manifestation of multi-target mechanisms of action by JLC, such as the inhibition of angiogenesis, immune modulation, and anti-inflammatory, antipyretic, and analgesic properties. These findings adequately demonstrate the multi-component, multi-target, and multi-pathway features of JLC, which is attuned to the complex biological characteristics of liver cancer. Therefore, the direction of future research should encompass the elucidation of the specific mechanisms of action of JLC and the exploration of ways to maximize its antitumor effects. Given the background of viral hepatitis and liver cirrhosis in most cases of liver cancer in China and the important role of the blood stasis syndrome in the occurrence of liver cancer, it is of utmost necessity to investigate the mechanisms of action of JLC, which possesses blood stasis removal and static blood dispersion functions. With the support of existing omics research and network pharmacology methodologies, elucidation of the material basis of the antitumor mechanisms of JLC has become highly possible. Based on definite mechanisms of action, clinical studies with a rigorous design and the effective and complementary combination of TCM and Western medicine approaches enable the targeting of benefiting populations and enhancement of clinical therapeutic effects.

The use of fresh medicinal ingredients for disease treatment is a prominent feature of TCM. JLC, an original drug produced from fresh medicinal animal materials by a unique process, is a Chinese patent drug used for the treatment of liver cancer that is attuned to the complex biological characteristics of liver cancer. It harnesses the unique and unconventional advantages of TCM and focuses on the clinical needs of liver cancer. Applying JLC in future research can further enhance evidence-based research on the TCM-based prevention and treatment of primary liver cancer, the development of precise synergistic strategies that combine TCM with modern medical treatment methods, and the improvement of treatment outcomes.

## 5 Conclusion

Primary liver cancer, a heterogeneous and deadly malignancy, demands innovative therapies. JLC, a Chinese patent medicine, shows clinical benefits in prolonging survival, enhancing response rates, and modulating immunity when combined with modern treatments. Preclinical studies reveal mechanisms including tumor growth inhibition, angiogenesis suppression, and immune regulation. Future research should clarify its molecular pathways, design rigorous trials with long-term survival endpoints and precise population stratification to develop synergistic strategies integrating JLC with modern medicine, providing new directions for liver cancer prevention and treatment.

## Abbreviations

5-FU, fluorouracil; CI, confidence interval; DFS, disease-free survival; FDA, Food and Drug Administration; HCC, hepatocellular carcinoma; JLC, jinlong capsule; mOS, median OS; NMPA, National Medical Products Administration; OR, Odds ratio; ORR, objective response rate; OS, overall survival; PD, programmed cell death; QOL, quality of life; RFA, radiofrequency ablation; TACE, transarterial chemoembolization; TCM, traditional Chinese medicine; VEGF, vascular endothelial growth factor; VEGFR2, VEGF receptor-2.
